# Blood pressure elevations post-lenvatinib treatment in hepatocellular carcinoma: a potential marker for better prognosis

**DOI:** 10.1038/s41440-025-02149-4

**Published:** 2025-02-18

**Authors:** Keisuke Shibata, Yuichi Akasaki, Akihiro Tokushige, Mina Nitta, Shin Kawasoe, Takuro Kubozono, Kohei Oda, Kotaro Kumagai, Seiichi Mawatari, Mitsuru Ohishi

**Affiliations:** 1 Department of Cardiovascular Medicine and Hypertension, Graduate School of Medical and Dental Sciences, Kagoshima University, Kagoshima, Japan; 2 Department of Prevention and Analysis of Cardiovascular Diseases, Graduate School of Medical and Dental Sciences, Kagoshima University, Kagoshima, Japan; 3 Department of Clinical Pharmacology and Therapeutics, Graduate School of Medicine, University of the Ryukyus, Okinawa, Japan; 4 Department of Clinical Pharmacy and Pharmacology, Graduate School of Medical and Dental Sciences, Kagoshima University, Kagoshima, Japan; 5 Digestive and Lifestyle Diseases, Kagoshima University Graduate School of Medical and Dental Sciences, Kagoshima, Japan

**Keywords:** Lenvatinib, Onco-Hypertension, VEGF Inhibitors

## Abstract

Lenvatinib is a tyrosine kinase inhibitor that effectively inhibits vascular endothelial growth factor signaling and is used for treating hepatocellular carcinoma. However, angiogenesis inhibitors often cause hypertension. Although lenvatinib-induced hypertension has been proposed as a potential surrogate marker for better prognosis, studies on blood pressure elevations and outcomes following lenvatinib initiation are limited. This study included 67 patients who underwent lenvatinib therapy at the Department of Gastroenterology, Kagoshima University Hospital, between May 2018 and December 2023. The median age of the cohort was 71 years, and 82.1% of the patients were male. The median blood pressure at admission was 128/73 mmHg, which significantly increased to 136/76 mmHg the day after lenvatinib administration. Grade 3 hypertension (≥160/100 mmHg) occurred in 37.3% of patients during hospitalization. The median increase in systolic blood pressure from admission to its peak during hospitalization was 26 mmHg. Patients who experienced an increase in blood pressure of ≥26 mmHg were classified into the blood pressure elevation group, which showed a significantly lower mortality rate than that of the blood pressure non-elevation group (35.3% vs. 81.8%, log-rank *p* = 0.007), even after adjusting for age, sex, disease stage, performance status, and liver reserve function. This study demonstrated that patients who experienced earlier blood pressure elevation after lenvatinib administration had lower overall mortality rates. These findings suggest that blood pressure elevations after lenvatinib initiation may serve as valuable prognostic indicators in patients with cancer undergoing lenvatinib therapy.

**Figure:**
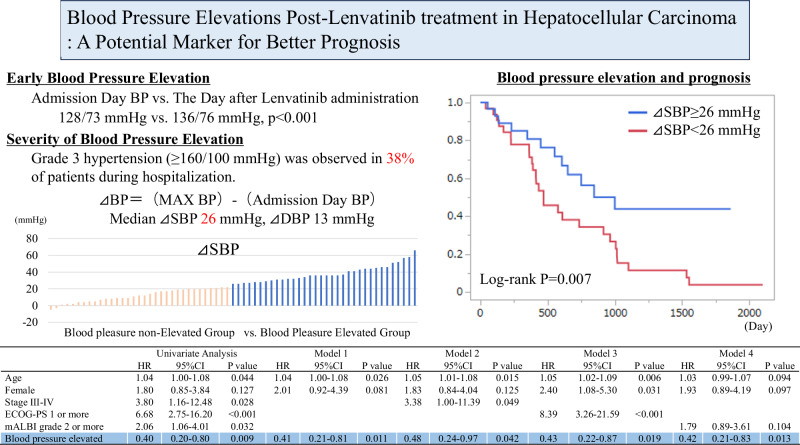
• Early Blood Pressure Elevation Following Lenvatinib Administration Significant blood pressure elevation was observed from the day after Lenvatinib administration, with a median systolic blood pressure increase of 26 mmHg. Grade 3 hypertension (≥160/100 mmHg) was observed in 38% of patients during hospitalization. •Blood Pressure Control Antihypertensive therapy was intensified in 39% of patients during hospitalization, yet 12% still had Grade 3 hypertension the day before discharge. • Association Between Blood Pressure Elevation and Prognosis Even after adjusting for age, sex, disease stage, performance status, and liver function reserve, blood pressure elevation was suggested as a better prognostic factor.

• Early Blood Pressure Elevation Following Lenvatinib Administration Significant blood pressure elevation was observed from the day after Lenvatinib administration, with a median systolic blood pressure increase of 26 mmHg. Grade 3 hypertension (≥160/100 mmHg) was observed in 38% of patients during hospitalization. •Blood Pressure Control Antihypertensive therapy was intensified in 39% of patients during hospitalization, yet 12% still had Grade 3 hypertension the day before discharge. • Association Between Blood Pressure Elevation and Prognosis Even after adjusting for age, sex, disease stage, performance status, and liver function reserve, blood pressure elevation was suggested as a better prognostic factor.

## Introduction

In an aging society, the number of patients with cancer is increasing. However, advancements in diagnosis and treatment have led to improved outcomes for many types of cancer. As the duration of therapy increases, the incidence of cardiovascular complications increases, drawing increased attention to the field of Onco-Cardiology.

Hypertension and its association with tumors have also gained attention, leading to an increased focus on Onco-Hypertension [1, 2]. Given that hypertension and cancer share common risk factors, such as smoking and salt intake, the incidence of cancer is notably higher among patients with hypertension, especially in men. Moreover, renal cell carcinoma and hypertension have been suggested to be associated with the onset of other malignancies [3–6]. Studies have been conducted from this perspective. A key area of interest in Onco-Hypertension is the increase in blood pressure owing to cancer pharmacotherapy, with hypertension induced by angiogenesis inhibitors being particularly well-known. In 1971, Folkman proposed the potential of angiogenesis inhibitors [7]. In 2004, bevacizumab, the first angiogenesis inhibitor, became available in the United States. Since then, several angiogenesis inhibitors have been introduced for cancer pharmacotherapy. These angiogenesis inhibitors have been reported to be associated with hypertension [8, 9]. The management of adverse effects during cancer pharmacotherapy was classified according to the Common Terminology Criteria for Adverse Events (CTCAE) grading system. In the CTCAE, the severity of adverse events is represented by Grades 1–5, where Grade 1 indicates mild, Grade 2 moderate, Grade 3 severe, Grade 4 life-threatening, and Grade 5 refers to mortality owing to adverse effects. For hypertension, Grade 3 is defined as blood pressure of ≥160/100 mmHg [10]. According to the CTCAE, when grade 3 or higher adverse effects are observed, dose reduction or discontinuation of cancer chemotherapy is required. Therefore, when using angiogenesis inhibitors, it is necessary to manage patients to prevent the occurrence of Grade 3 hypertension [11, 12].

In contrast, the incidence of hypertension following the administration of angiogenesis inhibitors has been suggested as a potential surrogate marker of a better prognosis [13]. We focused on lenvatinib, a tyrosine kinase inhibitor, and the associated hypertension. Lenvatinib is an oral multi-kinase inhibitor that targets receptors such as the vascular endothelial growth factor (VEGF) receptor and fibroblast growth factor receptor. In the international phase III trial (Resource Use of Lenvatinib Versus Sorafenib in First-Line Treatment of Hepatocellular Carcinoma [REFLECT] trial) for hepatocellular carcinoma (HCC), lenvatinib was shown to be non-inferior to sorafenib [14]. Consequently, lenvatinib has been approved for the treatment of “unresectable HCC” in Japan since March 2018. In the REFLECT trial, reports indicated that the incidence of all grades of hypertension was 39.7% and that of Grade 3 (≥160/100 mmHg) or higher hypertension was 22.1%. Among the Japanese population, hypertension occurred early and frequently, with an incidence of 49.4% for all grades and 32.1% for Grade 3 or higher hypertension [15]. Additionally, a relationship between hypertension and prognosis has been reported for lenvatinib. A subgroup analysis of the international phase III trial (Study of (E7080) lenvatinib in Differentiated Cancer of the Thyroid [SELECT] trial) for thyroid cancer revealed improved overall survival in patients who developed Grade 2 or higher hypertension after lenvatinib treatment compared with those who did not receive the treatment [16].

However, few studies have examined the details of early blood pressure elevation after lenvatinib administration or its association with prognosis. Therefore, this study aimed to clarify the patterns of blood pressure elevation after lenvatinib administration in Japanese patients with HCC and elucidate the association between early blood pressure elevation and prognosis.

## Methods

### Study design and patients

**Fig. 1:**
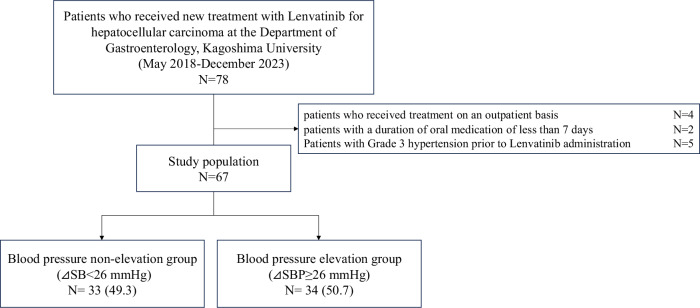
Study flow chart. Among the 78 patients who received their first administration of lenvatinib between May 2018 and December 2023, 67 were selected for analysis after excluding those treated on an outpatient basis, those who received oral administration for less than 7 days, and those with Grade 3 hypertension (≥160/100 mmHg) at the initiation of treatment. The group with a median increase in systolic blood pressure (SBP) of 26 mmHg or more, calculated as the highest systolic blood pressure during the hospitalization minus the systolic blood pressure on admission, was defined as the blood pressure elevation group. This group was compared with the blood pressure non-elevation group, which had an increase in SBP of less than 26 mmHg, in terms of patient background and prognosis

Among the 78 consecutive patients who received their first administration of lenvatinib at the Department of Gastroenterology, Kagoshima University Hospital, between May 1, 2018, and December 31, 2023, 67 are selected for analysis after excluding those who are treated on an outpatient basis (*n* = 4), those who received oral administration for less than 7 days (*n* = 2), and those with Grade 3 hypertension before treatment initiation (*n* = 5) (Fig. 1).

### Clinical data collection

Retrospective data were collected from electronic medical records, including basic patient information (age, sex, height, weight, body mass index [BMI], lifestyle history (smoking history, habitual alcohol consumption), medical history (hypertension, dyslipidemia, diabetes), laboratory findings before lenvatinib administration (peripheral blood, liver and kidney functions, glucose and lipid metabolisms), initial lenvatinib dosage, background liver condition, and tumor-related factors (stage, Eastern Cooperative Oncology Group Performance Status [ECOG-PS]) [17], as well as treatment history for HCC (surgery, trans arterial chemoembolization [TACE], radiofrequency ablation [RFA], history of pharmacotherapy).

### Definitions

Hypertension was defined according to the CTCAE version 5.0 [10]. Grade 1 hypertension was defined as systolic blood pressure (SBP) of 120–139 mmHg or diastolic blood pressure (DBP) of 80–90 mmHg, Grade 2 as SBP of 140–159 mmHg or DBP of 90–99 mmHg, and Grade 3 as SBP of 160 mmHg or higher or DBP of 100 mmHg or higher. In this study, blood pressure values that did not meet the criteria for Grade 1 hypertension were classified as Grade 0 for convenience. Pre-existing conditions before lenvatinib administration were defined as follows. Diabetes was defined as the requirement for medications (regular subcutaneous injections of insulin or glucagon-like peptide-1 inhibitors, or oral hypoglycemic agents), HbA1c of ≥6.5%, random blood glucose of ≥200 mg/dL, or fasting blood glucose of ≥126 mg/dL. Dyslipidemia was defined as the requirement for medications (regular subcutaneous injections of proprotein convertase subtilisin/kexin type 9 inhibitors or oral medication) or having total cholesterol of ≥220 mg/dL, triglycerides of ≥150 mg/dL, low-density lipoprotein cholesterol of ≥140 mg/dL, or high-density lipoprotein cholesterol of ≤40 mg/dL. Chronic kidney disease was defined as an estimated glomerular filtration rate of <60 mL/min/1.73 m^2^. Surgery was defined as any procedure performed under general anesthesia for HCC before lenvatinib initiation. BMI was calculated as weight (kg) divided by height squared (m^2^). The ECOG-PS score at admission was evaluated based on the ECOG criteria [17]. The modified albumin–bilirubin (mALBI) score was used to assess liver reserve function. The mALBI score was calculated using the following formula: log_10_ (total bilirubin [mg/dL] × 17.1) × 0.66 + (albumin [g/dL] × 10 × −0.085). The mALBI score was categorized as follows: Grade 1 (good liver function), ≤ −2.6 points; Grade 2a (moderate liver function), > −2.6 to < −2.27 points; Grade 2b (moderate liver function), ≥ −2.27 to ≤ −1.39 points; and Grade 3 (poor liver function), > −1.39 points [18]. The first day of lenvatinib administration was defined as Day 1.

### Blood pressure measurements and outcomes

We evaluated the changes in blood pressure after lenvatinib administration and the relationship between elevated blood pressure and survival rates. Blood pressure measurements and the corresponding CTCAE version. 5.0 grades were recorded for the highest blood pressure values observed on the day of admission, Day 2, Day 5, the day before discharge, and maximum values during hospitalization. The frequency of blood pressure measurements was determined at the discretion of the attending physician. Blood pressure was primarily measured in a seated position by nurses using the oscillometric method with the Elemano2 device (TERUMO Corporation, Tokyo, Japan). It was measured twice a day, often between 8:00–10:00 a.m. and 4:00–6:00 p.m. Additional blood pressure measurements were obtained as needed when high blood pressure was observed.

If Grade 3 hypertension was observed, the specific day on which it first occurred was recorded. Additionally, any increase in dosage or initiation of new antihypertensive medications during hospitalization was investigated. The following information was collected during the observation period, up to April 2024: we investigated whether lenvatinib was discontinued and, if so, we assessed the date of and reason for discontinuation. We also examined the occurrence, causes, and date of mortality from electronic medical records. Patients were categorized into two groups based on the median value of SBP, calculated as the difference between the highest SBP during hospitalization and the highest SBP on the day of admission. Patient backgrounds and outcomes were compared between the two groups (Fig. 1).

### Statistical analysis

Nominal variables are presented as numbers and percentages, and continuous variables as medians with interquartile ranges (IQR). The groups were compared using the chi-squared test for nominal variables and the t-test for continuous variables. Cumulative incidence was estimated using the Kaplan–Meier method, with differences evaluated using the log-rank test. A multivariate Cox proportional hazards analysis was conducted, with overall mortality as the outcome variable. Blood pressure elevation and clinically significant variables were included as covariates to assess the mortality risk. In all cases, a two-sided *p* < 0.05 was considered statistically significant. Statistical analyses were performed using JMP (version 17.0; Statistical Analysis System Institute, Cary, North Carolina, United States of America).

### Ethical considerations

Medical information was anonymized to ensure patient confidentiality. The Clinical Research Ethics Committee of Kagoshima University Hospital approved this study (approval number 230115), which was conducted in accordance with the Ethical Guidelines for Clinical Research of the Ministry of Health, Labor and Welfare of Japan and the Declaration of Helsinki. Participants provided informed consent using an opt-out approach.

## Results

### Baseline characteristics of the patients

The median age of the overall cohort was 71 (IQR 66–80) years, with 82.1% of the patients being male. The median BMI was 23.1 (IQR 20.3–26.0) kg/m². Hypertension, dyslipidemia, and diabetes were observed in 59.7%, 38.8%, and 38.8% of patients, respectively. The distribution of HCC was stages II, III, and IV in 13.4%, 41.8%, and 44.8% of patients, respectively, with 13.4% of patients having an ECOG-PS of 1 or higher. The background liver conditions include hepatitis B, hepatitis C, and non-B non-C in 22.4%, 25.4%, and 52.2% of patients, respectively. Treatment history included surgery in 31.3%, TACE in 74.6%, and RFA in 20.9% of patients, with 23.9% of patients having a history of pharmacological treatment for HCC (with overlap). The initial lenvatinib dose distribution was 4 mg, 8 mg, and 12 mg in 17.9%, 49.3%, and 32.8% of patients, respectively (Table 1).

**Table 1:** Baseline characteristics in all patients and according to elevated blood pressure

	Overall	Blood pressure non-elevation group (⊿SBP < 26 mmHg)	Blood pressure elevation group (⊿SBP ≥ 26 mmHg)	*P* value
	*n* = 67	*n* = 33	*n* = 34	
Age (years)	71 (66–80)	71 (66–80)	72 (67–77)	0.886
Male sex	55 (82.1)	27 (81.8)	28 (82.4)	0.955
BMI (kg/m^2^)	23.1 (20.3–26.0)	24.8 (21.0–26.8)	22.1 (20.3–25.5)	0.137
Smoking history	46 (68.7)	21 (63.6)	25 (73.5)	0.383
Habitual drinking history	43 (64.2)	20 (60.6)	23 (67.7)	0.548
Diabetes	26 (38.8)	10 (30.3)	16 (47.1)	0.159
Dyslipidemia	26 (38.8)	13 (39.4)	13 (38.2)	0.923
Hypertension	40 (59.7)	21 (63.6)	19 (55.9)	0.518
Antihypertensive medication	38 (56.7)	20 (60.6)	18 (52.9)	0.527
Baseline blood pressure				
Systolic (mmHg)	128 (116–136)	131 (124–138)	125 (114–132)	0.048
Diastolic (mmHg)	73 (62–80)	75 (62–79)	72 (64–81)	0.939
Hepatocellular carcinoma				
Stage				0.192
II	9 (13.4)	2 (6.1)	7 (20.6)	
III	28 (41.8)	14 (42.4)	14 (41.2)	
IV	30 (44.8)	17 (51.5)	13 (38.2)	
ECOG-PS				0.305
0	58 (86.6)	30 (90.9)	28 (82.4)	
1 or higher	9 (13.4)	3 (9.1)	6 (17.7)	
history of treatment for hepatocellular carcinoma				
surgical procedure	21 (31.3)	7 (21.2)	14 (41.2)	0.078
TACE	50 (74.6)	27 (81.8)	23 (67.7)	0.183
RFA	14 (20.9)	5 (15.2)	9 (26.5)	0.255
cancer pharmacotherapy	16 (23.9)	7 (21.2)	9 (26.5)	0.614
Etiology of chronic liver disease				0.714
Hepatitis B	15 (22.4)	6 (18.2)	9 (26.5)	
Hepatitis C	17 (25.4)	9 (27.3)	8 (23.5)	
non B non C	35 (52.2)	18 (54.6)	17 (50.0)	
Initial dose				0.767
4 mg	12 (17.9)	5 (15.2)	7 (20.6)	
8 mg	33 (49.3)	16 (48.5)	17 (50.0)	
12 mg	22 (32.8)	12 (36.4)	10 (29.4)	
Initial dose (mg/kg)	0.149 (0.120–0.174)	0.147 (0.118–0.176)	0.155 (0.118–0.173)	0.900
mALBI Grade 1	32 (47.8)	15 (45.5)	17 (50.0)	0.710
Baseline laboratory values				
White blood cell count (10^3^/μL)	5.3 (4.1–7.3)	5.5 (4.1–7.4)	5.0 (4.0–7.2)	0.701
Hemoglobin (g/dL)	13.0 (11.8–13.8)	13.5 (11.8–13.9)	12.9 (11.9–13.9)	0.587
Platelet count (10^2^/μL)	163 (108–207)	166 (116–207)	157 (105–208)	0.482
AST (U/L)	36 (27–53)	39 (26–56)	35 (26–47)	0.575
ALT (U/L)	22 (18–36)	22 (17–35)	22 (18–41)	0.861
LDH (U/L)	213 (182–261)	223 (190–290)	196 (168–250)	0.561
Albumin (g/dL)	3.8 (3.5–4.1)	3.8 (3.5–4.1)	3.8 (3.5–4.1)	0.573
Total bilirubin (mg/dL)	0.7 (0.6–1.0)	0.8 (0.6–1.0)	0.7 (0.5–1.1)	0.526
BUN (mg/dL)	15.3 (13.4–20.0)	15.3 (13.8–21.1)	15.6 (12.8–18.6)	0.244
Creatinine (mg/dL)	0.81 (0.69–0.94)	0.80 (0.70–0.99)	0.82 (0.69–0.93)	0.589
eGFR (mL/min/1.73 m^2^)	68.2 (58.8–82.2)	68.7 (56.3–85.2)	68.1 (61.9–78.5)	0.960
CKD	18 (26.9)	11 (33.3)	7 (20.6)	0.239
Total cholesterol (mg/dL)	172 (156–206)	170 (158–201)	174 (154–208)	0.957
Triglyceride (mg/dL)	88 (62–122)	88 (61–121)	88 (62–131)	0.796
High-Density Lipoprotein cholesterol (mg/dL)	56 (46–72)	53 (46–66)	58 (48–73)	0.423
Low-Density Lipoprotein cholesterol (mg/dL)	102 (77–128)	104 (78–130)	95 (77–127)	0.618
Blood glucose (mg/dL)	99 (89–116)	97 (88–102)	101 (90–131)	0.012
HbA1c (%)	5.8 (5.5–6.6)	5.7 (5.3–6.3)	6.0 (5.7–6.7)	0.034

*ALT* alanine aminotransferase, *AST* aspartate aminotransferase, *BMI* body mass index, *BUN* blood urea nitrogen, *CKD* chronic kidney disease, *ECOG-PS* Eastern Cooperative Oncology Group performance status, *eGFR* estimated glomerular filtration rate, *IQR* interquartile range, *LDH* Lactic Dehydrogenase, *mALBI* modified albumin‐bilirubin, *RFA*, Radiofrequency Ablation, *TACE* Transarterial Chemoembolization

### Blood pressure measurements and elevation in blood pressure after lenvatinib administration

**Fig. 2:**
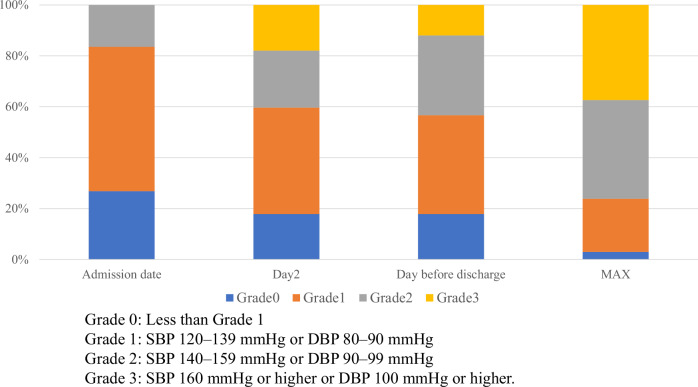
Histogram of hypertension grade classification according to the Common Terminology Criteria for Adverse Events for each day and the highest blood pressure during hospitalization. Distribution of blood pressure grades on the admission day, Day 2, the day before discharge, and the highest blood pressure during hospitalization. Grade 3 hypertension was observed in 17.9% of patients on Day 2, the day after starting lenvatinib, and in 11.9% of patients on the day before discharge. The highest blood pressure recorded during hospitalization was Grade 3 hypertension, noted in 37.3% of patients

**Fig. 3:**
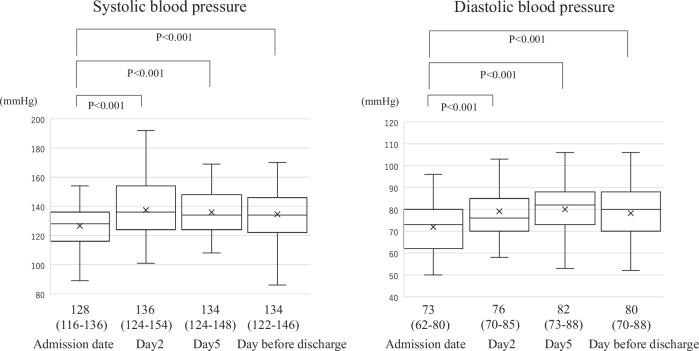
Blood pressure elevation after Lenvatinib administration. Blood pressure fluctuations before and after lenvatinib administration. A significant increase in blood pressure was observed on Day 2 than that on the admission day. However, no significant blood pressure fluctuations were noted on Day 5 and the day before discharge compared to those on Day 2

Measurements were generally instructed to be performed 2–3 times–once daily in 9 cases (13.4%), twice daily in 33 cases (49.3%), and thrice daily in 25 cases (37.3%). The median blood pressure upon admission was 128/73 mmHg. Grades 0, 1, and 2 were observed in 26.9%, 56.7%, and 16.4% of patients, respectively (Fig. 2). During the hospitalization period, 37.3% of patients experienced Grade 3 hypertension, with the median day of reaching Grade 3 hypertension being Day 3 (IQR 2–6) (Fig. 2). The median blood pressure on Day 2 was 136/76 mmHg, revealing a significant increase from that on the admission day (both *p* < 0.001). Blood pressure on Day 5 and the day before discharge remains significantly higher than that on the day of admission (Fig. 3). The median maximum blood pressure during the hospitalization period was 150/85 mmHg, with Grades 0, 1, 2, and 3 observed in 3.0%, 20.9%, 38.8%, and 37.3% of the patients, respectively (Fig. 2). The median difference between the maximum blood pressure and blood pressure on the day of admission indicates an increase of 26 mmHg in ΔSBP and 13 mmHg in ΔDBP. Overall, 38 patients (56.7%) were receiving antihypertensive medications at the time of lenvatinib initiation, including 3, 25, 31, 6, 4, and 1 patient(s) receiving angiotensin-converting enzyme inhibitor (ACE-i), angiotensin II receptor blockers (ARB), calcium channel blocker (CCB), β-blocker, antihypertensive diuretics, and mineralocorticoid receptor blockers (MRB), respectively (with some patients taking multiple drugs), respectively. During hospitalization, 38.8% of the patients experienced an increase in dosage or addition of other antihypertensive medications. Overall, 1, 8, 23, 0, 1, and 0 patient(s) required increased dosage or the addition of ACE-i, ARB, CCB, β-blockers, antihypertensive diuretics, and MRB (some patients were counted in multiple categories) (Supplemental Table 1). The decision to increase or add antihypertensive medications, as well as the choice of specific drugs, was made at the discretion of the attending hepatologist in this study. Despite the intensification of antihypertensive medications, Grade 3 hypertension was observed in 11.9% of the patients, even on the day before discharge.

### Discontinuation of lenvatinib and its reasons

The median observation period was 422 (IQR 165–915) days. During the observation period, lenvatinib was discontinued in 49 patients (73.1%). Regarding treatment discontinuation during the observation period, 17 patients (25.4%) were identified as being lost to follow-up. The median time to discontinuation was 231 (IQR 82–610) days. The main reasons for discontinuation were disease progression in 21 patients and gastrointestinal symptoms in 7 patients. None of the patients requires discontinuation owing to hypertension during the observation period.

### Blood pressure elevation and all-cause mortality

**Fig. 4:**
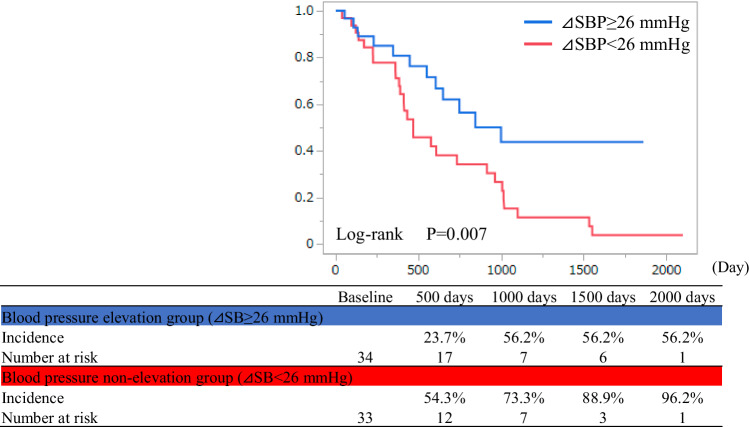
Kaplan-Meier curve of overall survival by elevated blood pressure. The Kaplan–Meier curves for all-cause mortality for the blood pressure elevation group (ΔSBP ≥ 26 mmHg) and blood pressure non-elevation group (ΔSBP < 26 mmHg). The blood pressure elevation group had significantly lower all-cause mortality (Log-rank *p* = 0.007)

During the observation period, 58.2% of the patients died, and 13 patients (19.4%) could not be tracked for mortality outcomes. The causes of death were cancer-related, cardiovascular-related, unknown in 32 (82.1%), 2 (5.1%), and 5 (12.8%) patients. A survival analysis based on baseline blood pressure grade showed no significant differences between the groups (log-rank *p* = 0.854) (Supplemental Fig. 1). Upon categorizing the patients into two groups based on whether the highest blood pressure during hospitalization was Grade 2 or higher versus Grade 1 or lower, no significant difference was observed (log-rank *p* = 0.493). However, upon categorizing the patients based on the median ΔSBP of 26 mmHg, those in the “blood pressure elevation group” (ΔSBP ≥ 26 mmHg) had significantly fewer mortalities compared to those in the “blood pressure non-elevation group” (blood pressure elevation group vs. blood pressure non-elevation group = 35.3% vs. 81.8%, *p* < 0.001, log-rank *p* = 0.007) (Fig. 4). The patient backgrounds were compared between the blood pressure elevation and non-elevation groups. No significant differences were observed in age, sex, BMI, history of hypertension treatment, diabetes, dyslipidemia, blood test results (peripheral blood, liver, renal function, and lipid metabolism), stage, ECOG-PS, liver reserve function (mALBI), treatment history for HCC, background liver condition, or initial lenvatinib dosage. However, the blood pressure elevation group had significantly lower systolic blood pressure before lenvatinib initiation (blood pressure elevation group vs. blood pressure non-elevation group = 125 (IQR 114–132)mmHg vs. 131 (IQR 124–138)mmHg, *p* = 0.048) and significantly higher blood glucose and HbA1c levels (Table 1). Cox proportional hazards analysis was performed, with mortality as the outcome variable. In univariate analysis, age, stage III or higher, ECOG-PS 1 or higher, and decreased liver reserve function (mALBI Grade 2 or higher) significantly increased the hazard ratio (HR) for all-cause mortality. Conversely, blood pressure elevation (ΔSBP ≥ 26 mmHg) significantly reduces the HR for mortality. In models adjusted for age and sex (Model 1), and with additional adjustments for disease stage (Model 2), ECOG-PS (Model 3), and liver reserve function (Model 4), blood pressure elevation continued to significantly reduce the HR for mortality (Table 2).

**Table 2:** Univariate and multivariate analysis using Cox proportional hazards for the incidence of all-cause death

	Univariate Analysis			Model 1			Model 2			Model 3			Model 4		
	HR	95%CI	*P* value	HR	95%CI	*P* value	HR	95%CI	*P* value	HR	95%CI	*P* value	HR	95%CI	*P* value
Age	1.04	1.00–1.08	0.044	1.04	1.00–1.08	0.026	1.05	1.01–1.08	0.015	1.05	1.02–1.09	0.006	1.03	0.99–1.07	0.094
Female	1.80	0.85–3.84	0.127	2.01	0.92–4.39	0.081	1.85	0.84–4.04	0.125	2.40	1.08–5.30	0.031	1.93	0.89–4.19	0.097
Stage III-IV	3.80	1.16–12.48	0.028				3.38	1.00–11.39	0.049						
ECOG-PS 1 or more	6.68	2.75–16.20	<0.001							8.39	3.26–21.59	<0.001			
mALBI grade 2 or more	2.06	1.06–4.01	0.032										1.79	0.89–3.61	0.104
Blood pressure elevated	0.40	0.20–0.80	0.009	0.41	0.21–0.81	0.011	0.49	0.24–0.97	0.042	0.43	0.22–0.87	0.019	0.42	0.21–0.83	0.013

*CI* confidence interval, *ECOG-PS* Eastern Cooperative Oncology Group performance status, *HR* hazard ratio; mALBI, modified albumin-bilirubin

Among the 49 patients who discontinued lenvatinib, 31 (63.3%) died during the observation period. However, upon examining mortality based on whether lenvatinib was discontinued, no significant difference was observed between the groups (no discontinuation vs. discontinuation: 8 (44.4%) vs. 31 (63.3%), *p* = 0.166). Similarly, regarding the degree of blood pressure elevation, no significant difference was found between the groups (no discontinuation vs. discontinuation: 32 (IQR 19–42) mmHg vs. 22 (IQR 10–36) mmHg, *p* = 0.235), with a trend suggesting a smaller extent of blood pressure elevation with the discontinuation of lenvatinib.

Additionally, the fact that 38.8% of patients required increased dosage or addition of other antihypertensive medications during hospitalization could not be ruled out as a potential factor influencing mortality. We performed a survival analysis by categorizing patients into four groups based on whether blood pressure elevation occurred after lenvatinib administration and whether antihypertensive medications were added or its dosage was increased. However, no significant differences were observed between the groups (log-rank *p* = 0.065, Supplemental Fig. 2). No significant association was observed between increased dosage or the addition of other antihypertensive medications and mortality (HR 0.78, 95% CI 0.40–1.52, *p* = 0.471). Furthermore, a multivariate analysis was conducted using the presence of blood pressure elevation and the addition or increase of antihypertensive medications (Model 5), as well as a model including age and sex as covariates (Model 6). In both models, the HR for mortality in the blood pressure elevation group significantly decreased, but increased dosage or the addition of other antihypertensive medications did not have a significant impact on mortality (Supplemental Table 2).

## Discussion

### Early blood pressure elevations after lenvatinib administration

VEGF inhibitors are associated with hypertension in 20–90% of cases [8, 9]. The mechanisms underlying hypertension induced by VEGF inhibitors, such as lenvatinib, are not yet fully understood. However, several proposed mechanisms are commonly considered, including the “vasodilator and vasoconstrictor imbalance theory,” “peripheral vascular resistance theory,” and “renal impairment theory” [6, 19]. In the international Phase III REFLECT trial for HCC, Grade 3 hypertension was observed in 25.1% of the overall population and 32.1% of the Japanese population after lenvatinib administration, indicating a higher incidence of hypertension-related complications among Japanese patients. Additionally, the median time to onset of hypertension was 26.0 days in the overall population and 15.0 days in the Japanese population, indicating an earlier onset of hypertension among Japanese patients [14, 15]. A previous study indicated that blood pressure elevation began the day after lenvatinib administration in Japanese patients with thyroid cancer [20]. This study demonstrated a significant blood pressure elevation beginning the day after lenvatinib administration, with Grade 3 hypertension occurring in 37.3% of patients. These findings are consistent with those of previous studies.

### Association between blood pressure elevation after lenvatinib administration and a better prognosis

During the observation period, 58.2% of patients died, with 82.1% of the deaths being cancer-related. Additionally, compared with the blood pressure non-elevation group, the blood pressure elevation group exhibited a better prognosis even after adjusting for age, sex, disease stage, performance status, and liver reserve function. Lenvatinib was indicated for “unresectable HCC” meaning the patients had advanced HCC, which likely accounts for the high proportion of cancer-related mortality.

Several reports have highlighted the association between the appearance of adverse effects after the administration of angiogenesis inhibitors and prognosis. Hypertension after bevacizumab administration is associated with a better prognosis [13]. Additionally, decreased appetite and fatigue associated with sorafenib or lenvatinib are linked to a worse prognosis. In contrast, the occurrence of hypertension and hand-foot syndrome is associated with a favorable prognosis [16, 21–24]. Although some side effects may be surrogate markers for a better prognosis, the mechanisms underlying this potential association require further investigation. In addition, this study suggested an association between early blood pressure elevation following lenvatinib administration and better prognosis. Blood pressure measurement is a minimally invasive procedure. The ability to predict cancer prognosis early after lenvatinib administration through this minimally invasive evaluation method is considered unique and valuable. Furthermore, the mechanisms underlying the relationship between the onset of side effects and prognosis require further investigation.

### Blood pressure elevation, rather than the Grade of hypertension, is associated with prognosis

As previously mentioned, while several studies have examined the relationship between hypertension and prognosis using VEGF inhibitors, few have focused on the degree of blood pressure elevation. Many studies define hypertension as Grade 2 or higher. This study conducted a survival analysis by categorizing patients based on whether their highest blood pressure grade during hospitalization was Grade 2 or higher. However, no significant differences were observed between the groups (log-rank *p* = 0.493). A previous study comparing patients with and without hypertension after lenvatinib administration indicated that the hypertensive group had higher baseline blood pressure [20]. In other words, evaluating blood pressure based on its grades may be heavily influenced by baseline blood pressure, potentially leading to an insufficient assessment of drug-induced blood pressure elevation. Evaluations based on hypertension grades may lead to an underestimation of patients whose blood pressure increases but remains within the normal range [25]. While we believe that evaluating blood pressure by its grade is helpful for its management, focusing on the extent of blood pressure elevation—rather than on the actual measured values or grades—may provide a better assessment of drug effects when evaluating blood pressure after VEGF inhibitor administration. Further investigation is needed to determine the degree of blood pressure elevation that constitutes a significant response to drugs.

### Comparison between the blood pressure elevation and non-elevation groups

This study revealed significant differences in blood pressure, blood glucose levels, and HbA1c levels between the hypertensive and non-hypertensive groups, but no other intergroup differences were noted. Additionally, no association was found between the initial lenvatinib dose and elevated blood pressure. A study targeting Japanese patients with thyroid cancer reported a correlation between the dosage of lenvatinib and its blood concentration but found no difference in blood concentration between different grades of hypertension [26]. In this study, no significant difference was observed in the maximum systolic blood pressure minus the systolic blood pressure on the admission day (ΔSBP) when comparing different dosage groups (4 mg vs. 8 mg vs. 12 mg = 31 mmHg vs. 26 mmHg vs. 22 mmHg, *p* = 0.237). It remains unclear which patients are more prone to elevated blood pressure. Identifying the predictors of blood pressure elevation may help identify patients who are likely to benefit from lenvatinib treatment. This study suggests that blood pressure elevation is a potentially favorable prognostic factor, even after adjusting for age, disease stage, ECOG-PS, and liver reserve function. Stage, ECOG-PS, and liver reserve function are critical determinants of prognosis of patients undergoing lenvatinib treatment. The fact that blood pressure elevation remains a good prognostic factor, even after adjusting for these variables, has significant clinical implications [27, 28]. Hypertension is an early and recognizable side effect, and because blood pressure measurement is minimally invasive and easily performed, it holds high potential for clinical application.

### Blood pressure management

During hospitalization, 38.8% of patients had their antihypertensive medication added or increased. However, 11.9% of patients still had Grade 3 hypertension, even on the day before discharge, indicating that blood pressure management was not entirely adequate. Blood pressure should be managed to prevent it from progressing to Grade 3 hypertension (≥160/100 mmHg) during lenvatinib administration. This may be achieved by intensifying antihypertensive therapy or reducing or disrupting lenvatinib dosage [11, 12]. Considering hypertension as a surrogate marker of a favorable prognosis, the need for antihypertensive treatment is sometimes questionable. However, studies have indicated a risk of aortic dissection following the administration of VEGF inhibitors [29]. Additionally, a study involving patients with renal cell carcinoma treated with sunitinib reported that the use of antihypertensive medications does not lead to a worse prognosis [30]. Therefore, hypertension should be managed to remain below Grade 3.

Therefore, patient education is crucial for effective blood pressure management. This includes performing home blood pressure measurements, utilizing tools such as a “lenvatinib diary” to record data, and sharing information on how to respond to elevated blood pressure in advance. In the cardiology department, vital points must be considered before, during, and after lenvatinib administration. The European Society of Cardiology Onco-Cardiology Guidelines advise assessing cardiac function based on risk factors before starting treatment with VEGF inhibitors, including lenvatinib. These guidelines also establish hypertension treatment goals tailored to cancer conditions. They recommend intervention when home systolic blood pressure measurements are ≥140 mmHg, regardless of disease status [12]. Therefore, it is reasonable to evaluate blood pressure during cardiac function assessment before starting lenvatinib treatment. If the home systolic blood pressure exceeds 140 mmHg, it is appropriate to consider intensifying antihypertensive therapy before initiating lenvatinib treatment. In addition, adjustments to antihypertensive medications may be required during and after lenvatinib administration. The optimal antihypertensive drugs for managing hypertension following the administration of VEGF inhibitors are not fully understood. However, in patients with proteinuria, prioritizing the use of renin–angiotensin system inhibitors is advisable [6]. In this study, CCB emerged as the most commonly prescribed antihypertensive medication. Considering the significant increase in blood pressure, many patients are likely to require combination therapy with antihypertensive drugs. Collaboration efforts among oncologists, cardiologists, pharmacists, and nurses to manage blood pressure can help reduce the need for dose reduction or discontinuation of lenvatinib owing to hypertension, potentially contributing to improved oncological outcomes. During the hospitalization period, no case was referred to the cardiology department for treatments such as antihypertensive therapy. Therefore, it is necessary to establish a system for collaboration and build a team to address this.

### Study limitations

This study has some limitations. It is a single-center retrospective study with a small sample size; therefore, larger sample sizes are needed in the future. The frequency, timing, and method of blood pressure measurement have not been established, and blood pressure was not accurately assessed. Furthermore, we only observed blood pressure variations during hospitalization and did not assess long-term changes, the degree of blood pressure control, or their impact on prognosis.

### Future perspectives

The extent of significant blood pressure elevation associated with a better prognosis after VEGF inhibitor administration may vary depending on the specific drug and cancer type. However, further investigation is necessary to elucidate this relationship. Additionally, if we can predict blood pressure elevation, it may be help us to identify patients who are likely to benefit from lenvatinib before administration.

Currently, no standardized protocol for blood pressure measurement following VEGF inhibitor administration is available, and we believe it is necessary to establish one in the future.

## Conclusion

Significant blood pressure elevation was observed in patients with HCC starting the day after lenvatinib administration. During hospitalization, the median increase in systolic blood pressure was 26 mmHg, suggesting that a rise of 26 mmHg or more may be suggested as a potential improved prognostic factor.

## Supplementary information

Supplemental Table 1

Supplemental Table 2

Supplemental Figure 1

Supplemental Figure 2

Supplemental Figures Legends
