# Supplementary material for: Blood pressure elevations post-lenvatinib treatment in hepatocellular carcinoma: a potential marker for better prognosis
Source: Hypertens Res. 2025 Feb 18;48(4):1542–53. doi: 10.1038/s41440-025-02149-4 (PMC11972954; doi:10.1038/s41440-025-02149-4)
Supplement: Supplementary file 1 — Supplemental Table 1 [file 41440_2025_2149_MOESM1_ESM.docx]

Supplemental Table 1

Number of patients by type of antihypertensive medication added or increased before Lenvatinib administration and during the hospitalization period (including duplicates)

|  | Baseline | addition or increase |
| --- | --- | --- |
| ACE-i | 3 | 1 |
| ARB | 25 | 8 |
| CCB | 31 | 23 |
| β-blocker | 6 | 0 |
| antihypertensive diuretics | 4 | 1 |
| MRB | 1 | 0 |

ACE-i, Angiotensin-Converting Enzyme inhibitor; ARB, Angiotensin II Receptor Blockers; CCB, calcium channel blocker; MRB, Mineralocorticoid Receptor Blockers.
