# Supplementary material for: Blood pressure elevations post-lenvatinib treatment in hepatocellular carcinoma: a potential marker for better prognosis
Source: Hypertens Res. 2025 Feb 18;48(4):1542–53. doi: 10.1038/s41440-025-02149-4 (PMC11972954; doi:10.1038/s41440-025-02149-4)
Supplement: Supplementary file 2 — Supplemental Table 2 [file 41440_2025_2149_MOESM2_ESM.docx]

Supplemental Table 2. Univariate and multivariate analysis using Cox proportional hazards for the incidence of all-cause death.

|  | Univariate Analysis | | | Model 5 | | | Model 6 | | |
| --- | --- | --- | --- | --- | --- | --- | --- | --- | --- |
|  | HR | 95%CI | P value | HR | 95%CI | P value | HR | 95%CI | P value |
| Age | 1.04 | 1.00-1.08 | 0.044 |  |  |  | 1.04 | 1.01-1.08 | 0.025 |
| Female | 1.80 | 0.85-3.84 | 0.127 |  |  |  | 2.00 | 0.23-1.09 | 0.081 |
| Stage Ⅲ-Ⅳ | 3.80 | 1.16-12.48 | 0.028 |  |  |  |  |  |  |
| ECOG-PS 1 or more | 6.68 | 2.75-16.20 | <0.001 |  |  |  |  |  |  |
| mALBI grade 2 or more | 2.06 | 1.06-4.01 | 0.032 |  |  |  |  |  |  |
| Blood pressure elevated | 0.40 | 0.20-0.80 | 0.009 | 0.40 | 0.20-0.82 | 0.0012 | 0.42 | 0.20-0.085 | 0.015 |
| Addition or increase of antihypertensive medication | 0.78 | 0.40-1.52 | 0.471 | 0.98 | 0.49-1.95 | 0.954 | 0.90 | 0.45-1.81 | 0.777 |

CI, confidence interval; ECOG-PS, Eastern Cooperative Oncology Group performance status; HR, hazard ratio; mALBI, modified albumin-bilirubin.
