# Supplementary material for: Blood pressure elevations post-lenvatinib treatment in hepatocellular carcinoma: a potential marker for better prognosis
Source: Hypertens Res. 2025 Feb 18;48(4):1542–53. doi: 10.1038/s41440-025-02149-4 (PMC11972954; doi:10.1038/s41440-025-02149-4)
Supplement: Supplementary file 3 — Supplemental Figure 1 [file 41440_2025_2149_MOESM3_ESM.pdf]

Supplemental Figure 1. Kaplan-Meier curve of baseline blood pressure grade

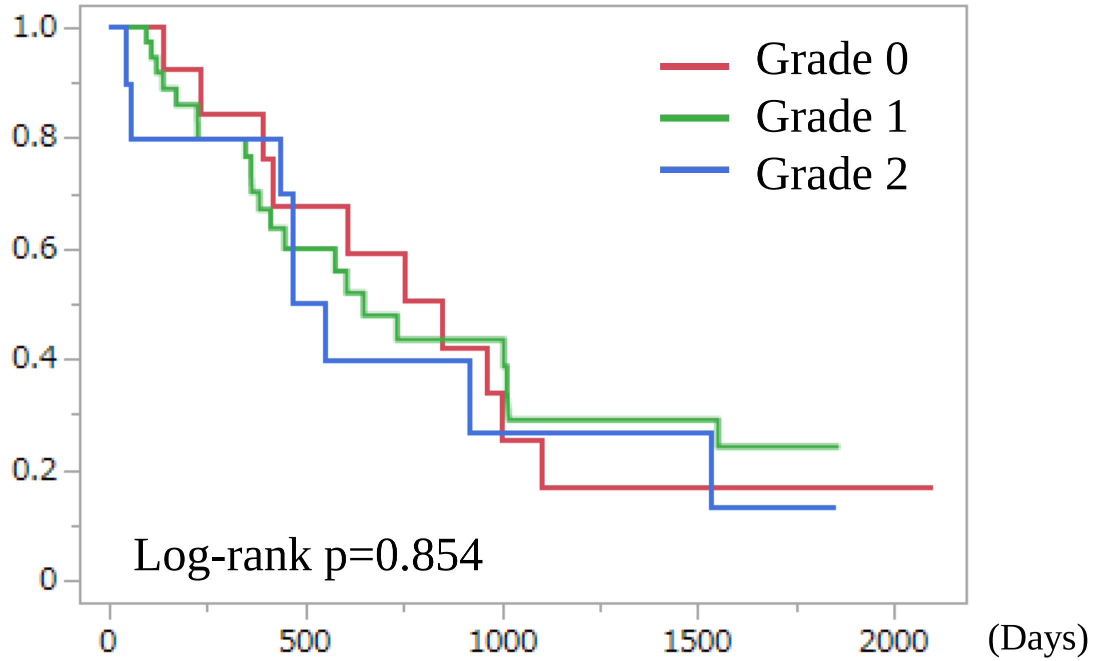

	Baseline	500 days	1000 days	1500 days	2000 days
Grade 0					
Incidence		32.3%	74.6%	83.1%	83.1%
Number at risk	18	9	4	3	2
Grade 1					
Incidence		40.1%	56.4%	71.0%	75.8%
Number at risk	38	17	10	7	1
Grade 2					
Incidence		50.0%	73.3%	73.3%	86.7%
Number at risk	11	7	3	3	1

Grade 0: Less than Grade 1
Grade 1: SBP 120–139 mmHg or DBP 80–90 mmHg
Grade 2: SBP 140–159 mmHg or DBP 90–99 mmHg
Grade 3: SBP 160 mmHg or higher or DBP 100 mmHg or higher.
