# Supplementary material for: Blood pressure elevations post-lenvatinib treatment in hepatocellular carcinoma: a potential marker for better prognosis
Source: Hypertens Res. 2025 Feb 18;48(4):1542–53. doi: 10.1038/s41440-025-02149-4 (PMC11972954; doi:10.1038/s41440-025-02149-4)
Supplement: Supplementary file 4 — Supplemental Figure 2 [file 41440_2025_2149_MOESM4_ESM.pdf]

Supplemental Figure 2. Kaplan-Meier curve of overall survival by elevated blood pressure and increase or addition of antihypertensive medication during hospitalization

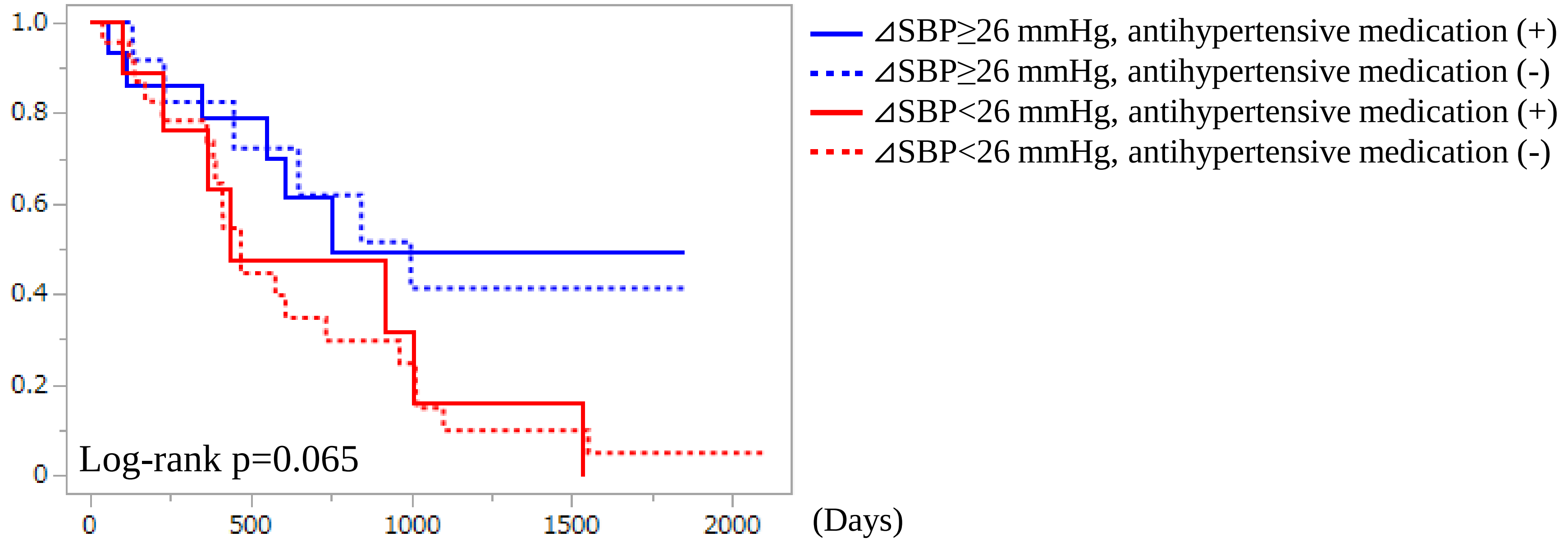

	Baseline	500 days	1000 days	1500 days	2000 days
⊿SBP≥26 mmHg, medication (+)					
Incidence		21.0%	50.9%	50.9%	50.9%
Number at risk	16	11	4	4	1
⊿SBP≥26 mmHg, medication (-)					
Incidence		27.8%	58.9%	58.9%	58.9%
Number at risk	18	8	5	5	1
⊿SBP<26 mmHg, medication (+)					
Incidence		52.4%	68.3%	84.1%	100.0%
Number at risk	10	4	3	2	1
⊿SBP<26 mmHg, medication (-)					
Incidence		55.4%	75.2%	90.1%	95.4%
Number at risk	23	11	6	3	2
