# Supplementary material for: Blood pressure elevations post-lenvatinib treatment in hepatocellular carcinoma: a potential marker for better prognosis
Source: Hypertens Res. 2025 Feb 18;48(4):1542–53. doi: 10.1038/s41440-025-02149-4 (PMC11972954; doi:10.1038/s41440-025-02149-4)
Supplement: Supplementary file 5 — Supplemental Figures Legends [file 41440_2025_2149_MOESM5_ESM.docx]

Supplemental Figure 1

The Kaplan-Meier curves of baseline blood pressure grade. No significant difference in prognosis was observed based on baseline blood pressure grade (Log-rank p = 0.854).

Supplemental Figure 2

The Kaplan-Meier curve of overall survival by elevated blood pressure and increase or addition of antihypertensive medication during hospitalization. The addition or increase of antihypertensive medication showed no significant association with prognosis (Log-rank p = 0.065).
